# Mechanisms of increased risk of tumorigenesis in *Atm *and *Brca1 *double heterozygosity

**DOI:** 10.1186/1748-717X-6-96

**Published:** 2011-08-17

**Authors:** Jufang Wang, Fengtao Su, Lubomir B Smilenov, Libin Zhou, Wentao Hu, Nan Ding, Guangming Zhou

**Affiliations:** 1 Key Laboratory of Heavy Ion Radiation Biology and Medicine, Institute of Modern Physics, Chinese Academy of Sciences, Lanzhou 730000, P. R. China; 2 Center for Radiological Research, Columbia University Medical Center, New York, NY 10032, USA; 3 Graduate School of Chinese Academy of Sciences, Beijing 100049, China

**Keywords:** heterozygosity, haploinsufficiency, tumorigenesis, DNA damage, cell cycle checkpoint, genomic instability

## Abstract

**Background:**

Both epidemiological and experimental studies suggest that heterozygosity for a single gene is linked with tumorigenesis and heterozygosity for two genes increases the risk of tumor incidence. Our previous work has demonstrated that *Atm/Brca1 *double heterozygosity leads to higher cell transformation rate than single heterozygosity. However, the underlying mechanisms have not been fully understood yet. In the present study, a series of pathways were investigated to clarify the possible mechanisms of increased risk of tumorigenesis in *Atm *and *Brca1 *heterozygosity.

**Methods:**

Wild type cells, *Atm *or *Brca1 *single heterozygous cells, and *Atm*/*Brca1 *double heterozygous cells were used to investigate DNA damage and repair, cell cycle, micronuclei, and cell transformation after photon irradiation.

**Results:**

Remarkable high transformation frequency was confirmed in *Atm*/*Brca1 *double heterozygous cells compared to wild type cells. It was observed that delayed DNA damage recognition, disturbed cell cycle checkpoint, incomplete DNA repair, and increased genomic instability were involved in the biological networks. Haploinsufficiency of either ATM or BRCA1 negatively impacts these pathways.

**Conclusions:**

The quantity of critical proteins such as ATM and BRCA1 plays an important role in determination of the fate of cells exposed to ionizing radiation and double heterozygosity increases the risk of tumorigenesis. These findings also benefit understanding of the individual susceptibility to tumor initiation.

## Background

Heterozygosity leads to haploinsufficiency for proteins. Heterozygous animals for one gene show similar appearance and life-span as the wild type in normal conditions since one of the alleles is still functional. However, heterozygous animals display high tumor incidence when exposed accidentally and severely to mutagens, such as high doses of ionizing radiation. It has been reported that *Pten *haploinsufficiency accelerated formation of astrocytomas and *CDKN1B *(also known as *p27*/*Kip1 *) haploinsufficiency contributed to leukemogenesis [1,2]. In general case, an individual may carry more than one heterozygous gene. Heterozygosity for two or more genes may multiply the risk of tumor initiation [3-5]. Double heterozygosity of *Pten/Trp53 *accelerated prostatic tumorigenesis [6]. A strong enhancement in mammary carcinogenesis by *Atm*/*p53 *double heterozygosity has also been reported [7].

It was found that genes critical for DNA damage signaling and repair pathways play an important role in tumorigenesis of heterozygosity [8-11]. ATM is both a sensor and transducer of DNA damage. The protein is rapidly activated by damage-induced autophosphorylation on ser^1981 ^followed by dissociation into an active monomer [12,13]. Once activated, it phosphorylates a series of downstream substrates including Nbs1, Chk1, Chk2, p53, etc., and finally activates cell cycle checkpoints, DNA repair as well as apoptosis [14]. It has been reported that *Atm *heterozygosity is linked with the induction of cataracts and tumorigenesis [15-18]. BRCA1 is also a multifunctional protein functioning in G2/M checkpoint and plays a central role in DNA repair [19,20]. The protein reduces the expression, phosphorylation and cellular localization of cdc25c and cdc2/cyclinB kinase, increases the expression of Wee1 kinase and 14-3-3σ to inhibit the G2-M transition. People who are heterozygous for *Brca1 *have a high risk of developing breast and ovarian cancer [21]. Contribution of *Brca1 *heterozygosity to tumor susceptibility has been reported [22,23].

In some cases, a multifunctional gene may be involved in more than one pathway when cells are exposed to exogenous stress. This makes the mechanisms of heterozygosity-related tumorigenesis more complicated. For example, both ATM and BRCA1 are part of BASC (BRCA1-associated genome surveillance complex), which is involved in recognition and repair of aberrant DNA structures [24,25]. DNA damage-induced ATM activation requires a coordinated assembly of BRCA1, BAAT1, and ATM [26], meanwhile BRCA1 is a substrate of ATM and its phosphorylation by ATM is critical for responding to double-strand breaks and G2/M checkpoint [27-29]. Epidemiological statistics reveals that reduction of ATM is common in BRCA1/2-deficient breast cancer [30]. Breast cancer in AT family is 1.5-14 folds higher [31-34]. Therefore, ATM and BRCA1 interact with each other and their association could be enhanced by mutagen treatment. Both of *Atm *and *Brca1 *are multifunctional genes involved in DNA repair pathways and cell cycle checkpoints. The risk of tumorigenesis may be increased in *Atm*/*Brca1 *double heterozygote.

Up to now, there are very few literatures on *Atm/Brca1 *double heterozygosity and their contribution to tumor initiation. In 2005, Bowen *et al*. reported that *Atm *heterozygosity increased the severity of mammary gland tumors in the BRCA1-defficient mouse [35]. Our previous work has demonstrated that double heterozygosity for *Atm *and *Brca1 *leads to higher cell transformation rate than single heterozygosity [36], but the underlying mechanisms have not been fully understood yet. In the present study, mouse embryonic fibroblast (MEF) cells of wild type, *Atm *or *Brca1 *single heterozygosity, and *Atm*/*Brca1 *double heterozygosity were used to develop the correlation of heterozygosity for *Atm *and/or *Brca1 *and tumorigenesis. A series of pathways including DNA damage recognition and repair, cell cycle checkpoints, genomic instability, and transformation frequency were investigated to identify the possible underlying mechanisms after the four genotypes of cells were exposed to photon radiation.

## Methods

### Cell culture

Four genotypes of MEF cells (wild type, ATMwt/BRCA1wt; *Atm *single heterozygote, ATMhz/BRCA1wt; *Brca1 *single heterozygote, ATMwt/BRCA1hz; and *Atm*/*Brca1 *double heterozygote, ATMhz/BRCA1hz) were obtained from the Center for Radiological Research, Columbia University Medical Center, New York. Cells were cultured with DMEM high glucose (Gibco, Grand Island, NY, USA) supplemented with 15% FBS (HyClone, Logan, UT, USA).

### Irradiation

A GammaCell 40 ^137^Cs irradiator (0.82 Gy/min) of Columbia University and a 6 MV X-ray generator (2 Gy/min) of Gansu Academy of Medical Sciences were used for the irradiation of the cells.

### Cell survival and neoplastic transformation

Anchorage-independent growth was conducted as described previously with slight modification [37]. Neoplastic transformation was assessed in a non-permissive condition of soft agar. Briefly, ϕ60 mm dishes were previously plated with 8 ml base agar layer prepared from a mixture of 68 ml 1.25% Bacto-agar, 17 ml tryptose phosphate broth (Sigma, St. Louis, MO, USA), 17 ml FBS and 68 ml double-strength of DMEM medium. After the mixture was solidified, 2.0 × 10^4 ^cells which had been exposed to 2 Gy of photons and incubated for one week were added to 2 ml of agar mixture prepared as above, and then plated on the agar base. Immediately after plating, cell clumps in each dish were marked and scored to avoid mis-counted as colonies. Cells were grown for 2 months in a CO_2 _incubator with saturated humidity. During this period 1 ml medium were added periodically to each dish. Colonies were counted as transformants using a dissecting microscope. For cell survival, similar procedure was followed but in a permissive condition. Specifically, 1.25% Bacto-agar was replaced with low-melting-point agarose and tryptose phosphate broth was replaced with distilled water. Cell survival was assessed right after cells were exposed to irradiation.

### γH2AX foci

Exponentially growing MEF cells of four genotypes were plated in four-well slide chambers (Lab-Tek, Naperville, IL, USA) at a density of 8 × 10^3 ^cells/well. Sixteen hours later, the cells were irradiated with 0.2 and 0.5 Gy of photons, fixed for 10 min in 4% paraformaldehyde, permeabilized for 5 min in methanol at -20°C, blocked for 1 h with 5% skim milk, and stained with mouse anti-γH2AX antibody (Upstate Biotechnology, Lake Placid, NY, USA) for 2 h. The bound antibody was visualized using Alexa Fluor^® ^488 anti-mouse antibody (Molecular Probes, Eugene, OR, USA), and cell nuclei were counterstained with PI/RNase solution (PharMingen, San Jose, CA, USA). Slides were observed under a confocal laser scanning microscope (Nikon, Tokyo, Japan). At least 100 cells were scored for each sample, and the average number of foci per cell was calculated.

### Comet Assay

DNA damage and repair were evaluated with alkaline comet assay according to the report of Olive *et al *with minor modifications [38]. In brief, single cells were harvested and re-suspended in DMEM medium containing 10% FBS at a concentration of 1 × 10^6 ^cells/ml. Cell suspension was mixed with 0.5% low melting-point agarose (Amresco, Solon, OH, USA) (1:3, V/V). The mixture was layered on each glass slide with pre-coated 0.5% LE agarose. After exposed to 10 Gy of photons, the samples were gently immersed into freshly prepared lysis solution (2.5 M NaCl, 10 mM Tris, 1% sodium lauryl sarcosinate, 100 mM EDTA, 1% Triton-100, and 10% DMSO) for 1.5 h and transferred to electrophoresis tank. After 20 min of DNA unwinding in buffer (1 mM EDTA, 300 mM NaOH, pH > 13), electrophoresis was performed in the same buffer (20 min, 20 V, 300 mA). The samples were neutralized with 0.4 M Tris-HCl (pH 7.5) and air-dried after a brief fixation with 70% ethanol.

Individual cells were visualized with ethidium bromide staining and photographed under a fluorescence microscope. For each sample, 200 comets were analyzed with CASP software [39].

### Cell cycle

Cell cycle distribution was monitored with a flow cytometer as described previously [40]. MEF cells exposed to 5 Gy of photons were post-incubated for various times, harvested, fixed with 70% pre-chilled ethanol for over 24 h at -20°C, re-suspended in PBS, and treated with PI/RNase solution. Cell cycle distribution of 10,000 cells for each genotype was analyzed with CellQuest pre-stored in FACScan flow cytometer (Beckon-Dickinson, CA, USA).

### Micronuclei

Exponentially growing MEF cells of four genotypes were plated in 12-well plates at a density of 3 × 10^5 ^cells/well. For each well, 3 μg/ml of cytochalasin B (Sigma, St. Louis, MO, USA) was added after cells were exposed to 2 Gy of photons. Twenty-four hours later, cells were washed with PBS and fixed with methanol-glacial acetic acid (9:1, V/V). After stained with 3 μg/ml Acridine Orange, at least 500 of binucleated cells for each genotype were counted to obtain the frequency of micronuclei.

### Statistical Analysis

All data were presented as mean ± SE of at least three independent experiments. Students' *t*-test was used to evaluate statistically significant.

## Results

### Transformation

**Figure 1:**
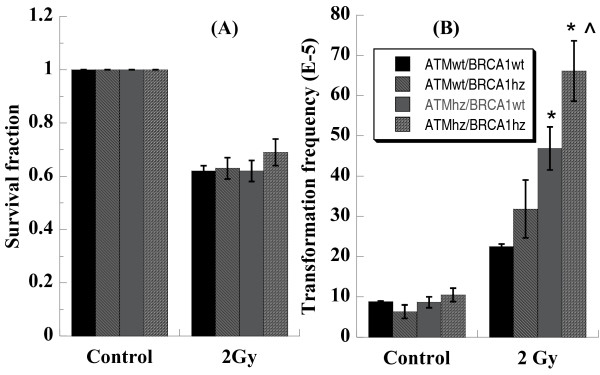
**Anchorage-independent growth levels of four kinds of MEF cells**. After cells were exposed to 2 Gy of photons, permissive soft-agar assay was performed immediately to assess cell survival (Panel A) and 1 week later, non-permissive soft-agar assay were performed to assess neoplastic transformation (Panel B). Transformants were visually scored under a dissecting microscope. Data are from four independent experiments. * indicates the significant difference (p < 0.05) compared with ATMwt/BRCA1wt cells and ^ indicates the significant difference (p < 0.05) compared with ATMwt/BRCA1hz cells experienced the same treatment.

Anchorage-independent growth assay was carried out to measure cell survival of four kinds of MEF cells in permissive condition right after the cells were exposed to 2 Gy of photons and to measure neoplastic transformation in non-permissive condition 1 week after irradiation. Results showed that 2 Gy of photons inactivated the four kinds of MEF cells almost equally (Figure 1A). However, induced cell transformation was in a genotype-dependent manner (Figure 1B). For sham irradiated sample, an average transformation frequency of 8.86 ± 0.14, 6.31 ± 1.66, 8.69 ± 1.35, and 10.50 ± 1.64 (× 10^-5^, transformants per 10^5 ^viable cells) was obtained for ATMwt/BRCA1wt, ATMwt/BRCA1hz, ATMhz/BRCA1wt, and ATMhz/BRCA1hz, respectively. No significant differences were observed. Upon photon irradiation, dramatically increased yields of anchorage-independent colonies were observed in the four kinds of cells in a genotype-dependent manner. Remarkably high transformation frequency was obtained in double heterozygous cells while wide type cells presented the lowest transformation frequency and single heterozygous cells presented the middle ones. This result confirms that double heterozygosity is confronted with higher tumorigenesis risk than single one.

### DNA damage and repair

In this study, we used two methods to observe DNA damage and repair ability of cells heterozygous for *Atm *and/or *Brca1*. One was γH2AX focus formation by immunofluorescent staining to visualize DSBs [41] and the other was alkaline comet assay to quantify total DNA damage.

**Figure 2:**
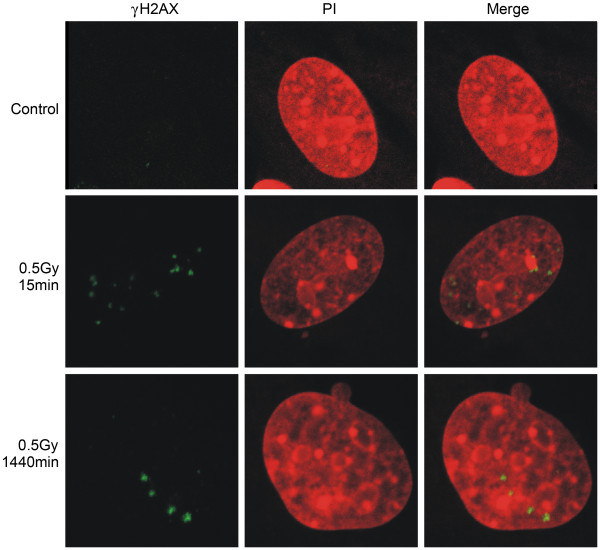
**Representative pictures of γH2AX foci**. Cells were exposed to 0.5 Gy of photons and stained with mouse anti-γH2AX antibody visualized by Alexa Fluor^® ^488 anti-mouse antibody and propidium iodide (PI) at 15 and 1440 min post-irradiation.

**Figure 3:**
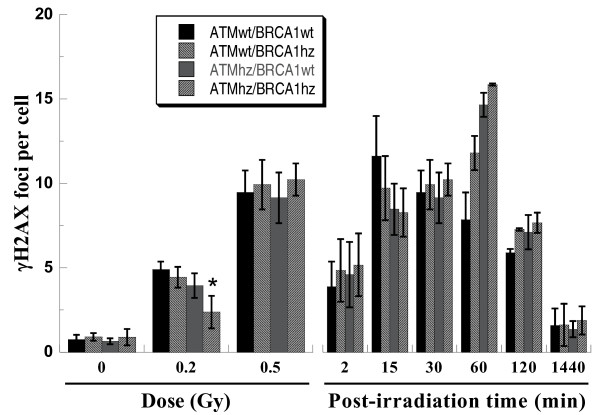
**Quantitative detection of DSBs by counting γH2AX foci**. Dose response (left) and kinetics (right) of γH2AX focus formation. Samples for dose response were fixed at 30 min after irradiation and kinetics was for 0.5Gy of photons. * indicates the significant difference (p < 0.05) compared with ATMwt/BRCA1wt cells exposed to 0.2 Gy of photons.

Representative pictures of γH2AX foci were shown as Figure 2. Consistent with other reports, γH2AX foci were formed in a dose- and time- dependent manner (Figure 3). The dose response of γH2AX focus formation was scored at 30 min post-irradiation. The foci per cell induced by 0.5 Gy of photons did not show statistical differences in all kinds of MEF cells. However, significant differences were observed when cells were exposed to 0.2 Gy of photons. The lowest number was in double heterozygous cells. This genotype-dependent manner was confirmed by the kinetics of focus formation. The number of γH2AX foci increased very rapidly after irradiation even during the very first 2 min required for sample preparation. The number of foci in wild type cells reached to the maximum at 15 min and then decreased gradually. *Atm *single heterozygous cells, *Brca1 *single heterozygous cells and *Atm/Brca1 *double heterozygous cells spent as long as 60 min before the approach to the maximum. It indicates that the response of wild type cells to DSBs induced by ionizing radiation is faster than the response of heterozygous cells. The recognition of DNA damage is delayed in heterozygote.

**Figure 4:**
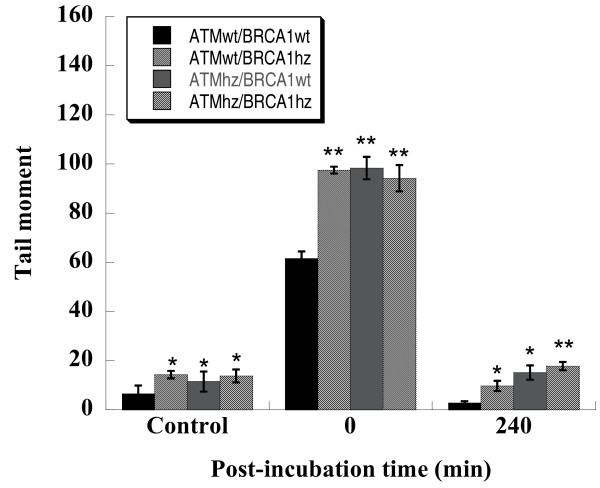
**Total DNA damage measured with alkaline comet assay in four kinds of MEF cells at different time course**. Data were a pool of three independent experiments and totally 200 cells were scored. * and ** indicate the significant difference compared with ATMwt/BRCA1wt cells experienced the same treatment and same post-incubation (* for p < 0.05 and ** p < 0.01).

In previous studies, we observed significantly higher background of DNA damage in heterozygous cells [36]. We observed the induction and repair of DNA damage induced by photon irradiation in this study with the same technology, comet assay. As shown in Figure 4, without post-irradiation incubation, DNA damage induced in wild type cells was significantly lower than that in other three kinds of cells (p < 0.01). It might be due to the rapid repair processes that occurred during comet assay preparation. At 240 min post-incubation, DNA damage in each cell type decreased to background level. However, the residual DNA damage in double heterozygous cells was the highest one among the four kinds of MEF cells, and the residual DNA damages in single heterozygote were also significantly higher than that in wild type cells. These results suggest that DNA repair may be delayed in heterozygote and DNA repair ability may be the lowest in double heterozygote.

### Cell cycle dynamics

**Figure 5:**
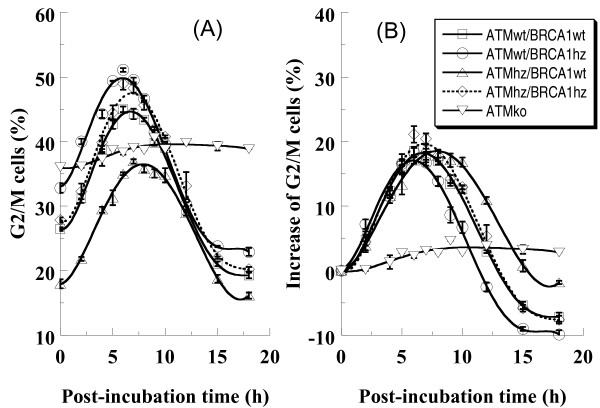
**Cell cycle distribution in MEF cells exposed to 5 Gy of photons**. Kinetics of G2/M block based on original data (Panel A) and optimized dada in which the control level of G2/M-phase cells was correspondingly subtracted from the proportion of G2/M-phase cells of each sample (Panel B). Data are pooled from three independent experiments.

As expected, 5 Gy of photons induced G2/M block in all genotypes. Notably the induction of G2/M block was similar in all the genotypes (Figure 5). However, different genotypes followed different dynamics of G2/M-phase cell release. Wild type cells overcame the G2/M arrest in 13 hours. *Brca1 *single heterozygous cells overcame that block earlier while *Atm *single heterozygous cells later. Interestingly, single heterozygosity results in disturbed cell cycle control while double heterozygous cells showed a similar cell cycle dynamics to the wild type cells.

### Genomic instability

**Figure 6:**
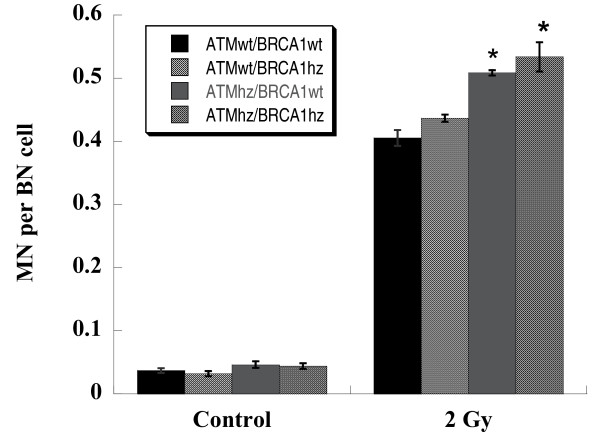
**Induction of micronuclei in MEF cells exposed to 2 Gy of photons**. Individual cells were visualized with Acridine Orange staining and micronuclei (MN) per binucleated cell (BN) were score under fluorescence microscope. More than 500 BN cells were counted for each sample. Data were pooled from three independent experiments. * indicates the significant difference (p < 0.05) compared with ATMwt/BRCA1wt cells exposed to the same dose of photons.

Cytochalasin-blocked micronucleus assay is a widely used method for the detection of genomic instability. The micronuclei per binucleated cell induced by 2 Gy of photon in each kind of cells were not the same (Figure 6). The highest micronucleus frequency was observed in double heterozygous cells while the lowest in wild type cells. This result demonstrates that micronucleus formation can be obviously enhanced in *Atm/Brca1 *double heterozygous cells, suggesting an impairing of genomic stability.

## Discussion

Heterozygosity results in haploinsufficiency. Haploinsufficient animals or cells for DNA-repair related proteins appear normal under stationary conditions. However, high tumor incidence or transformation rate is observed when they are exposed severely and accidentally to exogenous stimuli. It is very possible that protein products from one allele are not enough for the detection and repair of damage induced by the exogenous factors. On the other hand, cell cycle checkpoint is activated to earn time for damage repair. If the heterozygous gene takes part in the cell cycle checkpoint pathway, cell cycle checkpoint might also be negatively impacted and cells with un-repaired damage might escape the monitor and pass the remained lesions to their daughter cells, which have high potential for genomic instability, transformation, and consequently high risk of tumorigenesis.

### How does haploinsufficiency impact the processes of DNA damage recognition?

In 2006, Smilenov proposed a theory of temporal biological network to explain the mechanisms underlying the link between heterozygous *Atm *and/or *Rad9 *and tumor predisposition, in which a temporal network assembled by both gene products and DNA damage caused by ionizing radiation is thought to be critical to activate apoptosis pathways [42]. According to this theory, ATM-dependent local network is a critical element in a cell responding to DNA damage. The number of local networks assembled on the damage points determines the generated signal level, which in turn controls the induction of apoptosis. Theoretically, there is no problem for wide type cells to recognize DNA damage induced by high-dose and low-dose radiation. In the case of heterozygosity and high-dose radiation, heterozygous cells can recognize DNA damage due to enough DNA damage even though there are no enough gene products to work efficiently. However, in the case of heterozygosity and low-dose radiation, since neither gene products nor DNA damage is enough, limited number of local networks might be assembled to trigger apoptosis. Some of the damages will not be detected, and apoptotic response can not be fully activated. Heterozygous cells with lesions might survive the stimulation, which consequently results in tumor initiation.

In the present study, we employed γH2AX foci to observe the DNA recognition because it is a rapid process and sensitive method. After irradiation, γH2AX foci usually appear in 30 seconds and increase until a plateau is reached by 10-30 minutes [43]. The level of the plateau is proportional to radiation dose [44]. Along with further incubation, the number of foci per cell decreases due to DNA repair. Therefore, the height of plateau may reflect the original damage level, and the formation process of γH2AX foci may represent the recognition of DNA damage. As shown in Figure 3, the genotype-dependent difference of γH2AX foci implied that some DNA damage induced by 0.2 Gy photons could not be well recognized in double heterozygosity. Furthermore, it took heterozygous cells longer time to respond to radiation as confirmed by the time kinetics. Thus, Simlenov's hypothesis can be broadened to more biological processes. In the case of heterozygosity and low-dose radiation, no enough temporary biological networks are assembled to activate a series of pathways, such as damage recognition, DNA repair, apoptosis, and so on. Double heterozygosity leads to the alleviated assembly of local networks. One possible pathway of those heterozygous cells with un-repaired damage is to go to reproductive death, and another is to escape from cell cycle monitoring, apoptosis pathway, and consequently transfer the lesions to daughter cells through mitosis.

### How does haploinsufficiency impact cell cycle checkpoints?

ATM activates a series of DNA-repair related factors [27] and cell cycle checkpoints factors [14,45]. BRCA1 is one of the key proteins participating in a common pathway to facilitate orderly homologous recombination and thereby maintaining genomic stability [20]. Therefore, insufficient products of these two genes supposedly lead to incompletely functional cell cycle checkpoints.

In our experiments, *Atm*-knockout cells have a high proportion of G2/M phase, which is in consistent with the other report [46]. As shown in Figure 5, *Atm *knockout cells exposed to 5 Gy of photons had prolonged G2 arrest, which implies the important role of ATM in G2-M transition. Then, what is the possible consequence of the delay of G2-arrest release in cells heterozygous for *Atm*? Since ATM is a sensor detecting DNA damage and also a transducer activating downstream genes to repair the damage, its insufficient expression theoretically reduces the efficiency of DNA repair. The extra repair time obtained from the prolonged G2 arrest could not compensate this inefficiency, which can be concluded from the results of DNA repair measured by alkaline comet assay. As shown in Figure 4, the wild type cells have the most efficient repair ability, and the double heterozygote has the least. On the other hand, a very possible consequence of the abrogation of G2 arrest in cells heterozygous for *Brca1 *is that some cells with un-repaired DNA damage escape the cell cycle monitoring and transfer the damage to their offspring.

### *Atm *and *Brca1 *heterozygosity enhances transformation frequency and increases the risk of tumorigenesis

Since *Atm*/*Brca1 *double heterozygosity results in low apoptosis [36], a very possible consequence is that residual DNA damage escaped from cell cycle checkpoints arouses genomic instability in the offspring of heterozygous cells. As shown in Figure 6, micronuclei induced by photons were genotype-dependent. The highest micronucleus frequency was obtained in double heterozygote while the lowest in wild type cells.

High sensitivity to transformation and low level of DNA damage repair were reported in double heterozygote induced by radiation [36, 47 and 48]. Our results confirmed that heterozygosity of either *Atm *or *Brca1 *resulted in increased cell transformation and *Atm*/*Brca1 *double heterozygosity enhanced the transformation frequency significantly (Figure 1).

These results imply the positive correlation of genomic instability with cell transformation and the consequently high frequency of tumor initiation in double heterozygote.

## Conclusions

Taken together, haploinsufficiency of either ATM or BRCA1 negatively impacts a series of pathways including delayed DNA damage recognition, disturbed cell cycle checkpoint, incomplete DNA repair, increased genomic instability and transformation frequency. The quantity of critical proteins such as ATM and BRCA1 plays an important role in determination of the fate of cells exposed to ionizing radiation and *Atm*/*Brca1 *double heterozygosity increases the risk of tumorigenesis. These findings not only provide an experimental proof to Smilenov's hypothesis and broaden it to more biological processes, but also benefit understanding of the individual susceptibility to tumor initiation.

## List of abbreviations

*Atm*: ataxia telangiectasia mutated; *Brca1*: breast cancer 1; MEF: mouse embryonic fibroblast; SE: standard error; PCR: polymerase chain reaction; DMEM: Dulbecco's Modified Eagle Medium; FBS: fetal bovine serum; PBS: phosphate buffered saline.

## Competing interests

The authors declare that they have no competing interests.

## Authors' contributions

GZ and LBS conceived the study and participated in its design. LBS isolated and provided the heterozygous cells. FS, LZ, WT and ND carried out the γH2AX foci, comet, cell cycle, micronuclei and transformation assay. JF participated in coordination and drafted the manuscript. All authors read and approved the final manuscript.
